# Predictive Biomarkers and Resistance Mechanisms of Checkpoint Inhibitors in Malignant Solid Tumors

**DOI:** 10.3390/ijms25179659

**Published:** 2024-09-06

**Authors:** Luciana Alexandra Pavelescu, Robert Mihai Enache, Oana Alexandra Roşu, Monica Profir, Sanda Maria Creţoiu, Bogdan Severus Gaspar

**Affiliations:** 1 Department of Morphological Sciences, Cell and Molecular Biology and Histology, Carol Davila University of Medicine and Pharmacy, 050474 Bucharest, Romania; luciana.pavelescu@umfcd.ro (L.A.P.); oana-alexandra.rosu@rez.umfcd.ro (O.A.R.); monica.profir@rez.umfcd.ro (M.P.); 2 Department of Radiology and Medical Imaging, Fundeni Clinical Institute, 022328 Bucharest, Romania; robert-mihai.enache@rez.umfcd.ro; 3 Department of Oncology, Elias University Emergency Hospital, 011461 Bucharest, Romania; 4 Department of Surgery, Carol Davila University of Medicine and Pharmacy, 050474 Bucharest, Romania; bogdan.gaspar@umfcd.ro; 5 Surgery Clinic, Bucharest Emergency Clinical Hospital, 014461 Bucharest, Romania

**Keywords:** predictive biomarkers, immune checkpoint inhibitors, solid tumors, microbiota

## Abstract

Predictive biomarkers for immune checkpoint inhibitors (ICIs) in solid tumors such as melanoma, hepatocellular carcinoma (HCC), colorectal cancer (CRC), non-small cell lung cancer (NSCLC), endometrial carcinoma, renal cell carcinoma (RCC), or urothelial carcinoma (UC) include programmed cell death ligand 1 (PD-L1) expression, tumor mutational burden (TMB), defective deoxyribonucleic acid (DNA) mismatch repair (dMMR), microsatellite instability (MSI), and the tumor microenvironment (TME). Over the past decade, several types of ICIs, including cytotoxic T-lymphocyte-associated protein 4 (CTLA-4) inhibitors, anti-programmed cell death 1 (PD-1) antibodies, anti-programmed cell death ligand 1 (PD-L1) antibodies, and anti-lymphocyte activation gene-3 (LAG-3) antibodies have been studied and approved by the Food and Drug Administration (FDA), with ongoing research on others. Recent studies highlight the critical role of the gut microbiome in influencing a positive therapeutic response to ICIs, emphasizing the importance of modeling factors that can maintain a healthy microbiome. However, resistance mechanisms can emerge, such as increased expression of alternative immune checkpoints, T-cell immunoglobulin (Ig), mucin domain-containing protein 3 (TIM-3), LAG-3, impaired antigen presentation, and alterations in the TME. This review aims to synthesize the data regarding the interactions between microbiota and immunotherapy (IT). Understanding these mechanisms is essential for optimizing ICI therapy and developing effective combination strategies.

## 1. Introduction

ICIs offer a groundbreaking approach to treating various malignant solid tumors, contrasting with traditional cytotoxic therapies [1]. As immune cell surface receptors, ICIs have the capacity to either activate or inhibit the immune response. In the context of IT, they function as blockers of T-lymphocyte cell surface receptors, thereby enhancing the antitumor immune response [2].

For a long time, the progress in IT has been eclipsed by the rapid advancements in radiotherapy (RT) and chemotherapy [1,3]. However, recent research focused on cancer immunology and IT has enhanced our understanding of the antitumor response of T cells, as well as their interactions with cancer cells, antigen-presenting cells (APCs), immunosuppressive cells, and other immune mechanisms [1,3]. Studies by Bretcher and Cohn and Jenkins and Schwartz highlight that T-cell activation occurs via the T-cell receptor (TCR), which recognizes specific antigens, and through the costimulatory interaction between a T-cell costimulatory receptor and its ligand on an APC [4,5]. The inhibitory signals in T cells ensure tolerance to self-antigens, while the stimulatory signals promote an adaptive immune response to foreign antigens, collectively regulating immune activation [6]. Immune checkpoints allow inhibitory stimuli to modulate the inflammatory response following T-cell activation and protect normal cells from T-cell-mediated cytotoxicity [6]. This mechanism is crucial as it prevents the T-cell immune response from targeting cancer cells within the TME, serving as a protective measure [6].

Over the past decade, several types of ICIs have been studied and approved by the FDA. The first of these was the CTLA-4 inhibitor, Ipilimumab, approved in 2011 for metastatic malignant melanoma [2]. CTLA-4 exerts a negative regulatory effect on T cells, and its blockade can inhibit tumor formation, making it useful in cancer IT [2]. Additionally, the FDA has approved three anti-PD-1 antibodies—Pembrolizumab, Nivolumab, and Cemiplimab—and three anti-PD-L1 antibodies—Atezolizumab, Durvalumab, and Avelumab [1]. PD-1 and PD-L1 induce T-cell exhaustion, and blocking these pathways has been shown to enhance tumor-specific T-cell responses and promote tumor regression [2]. Ongoing research is exploring other potential ICI targets, including inhibitory receptors like TIM-3, V-domain Ig suppressor of T-cell activation (VISTA), and LAG-3, as well as activating molecules such as OX40 (CD134) and glucocorticoid-induced TNFR-related protein (GITR) [2]. However, studies have yielded mixed results, with some patients experiencing sustained therapeutic responses and others showing diminished responses over time [7]. This variability has highlighted the need to identify molecular biomarkers to predict treatment response and resistance mechanisms to checkpoint inhibitors, which will be discussed further in this article.

Malignant cells possess immunosuppressive properties, such as the expression of PD-L1 and the secretion of suppressive cytokines, which help them reduce their immunogenicity [8]. The resistance mechanisms of malignant cells can be categorized into two main strategies: evading immune recognition and creating an immunosuppressive TME [8]. These mechanisms rely on tumor cell-intrinsic and -extrinsic factors that interfere with antigen processing and presentation or enable tumors to recruit immune-suppressing cells that counteract the activity of effector cells [9]. Recent studies highlight the pivotal role of the TME in tumor-induced immunosuppression, as it is involved in downregulating effector T-cell activity and recruiting immunosuppressive cells [10].

Biomarkers can be categorized as either predictive or prognostic markers. Predictive markers, like the BRAF mutation status in melanoma, help determine the likelihood of a patient’s response to specific treatments, such as BRAF/MEK inhibitors [11,12]. Prognostic markers, such as the overall stage of disease in solid cancers and prostate-specific antigen levels at prostate cancer diagnosis, are independent of cancer interventions and can predict disease recurrence or patient survival [11,13]. The hormonal status of breast cancer serves a dual purpose as both a prognostic and predictive biomarker. Recent studies suggest that ongoing research and a deeper understanding of biological processes will enhance existing biomarkers and aid in developing new ones. PD-L1 expression and MSI are the two FDA-approved biomarkers used with ICIs. However, these biomarkers have shown limitations. Due to the genomic variability of humans and tumor cells, future approaches will likely require the use of multiple biomarkers to predict responses to ICIs accurately [11].

Recently, significant interest has emerged in studying the role of the gut microbiota in shaping responses to ICIs [11]. Several studies have identified bacterial species linked to favorable responses to ICIs, such as *Akkermansia muciniphila*, and others associated with resistance, like *Ruminococcus obeum* [11,14,15]. In patients with NSCLC or RCC, the use of antibiotics during ICI initiation has been associated with reduced microbiome diversity, leading to decreased overall survival and progression-free survival [14,16]. Additionally, a high-fiber diet has been shown to have a more beneficial effect on ICI response than a low-fiber diet [16].

## 2. Immune Checkpoint Inhibition in Cancer—Mechanisms of Action and Resistance Mechanisms

A class of medications known as ICIs is used to treat cancer [17]. These medications stimulate the body’s immune system to target cancer cells more successfully. It is useful to have some understanding of the interactions between cancer cells and the immune system to comprehend how they function [17].

The immune system’s mission is to identify aberrant cells, including cancerous ones, and eliminate them. Immune cells, known as T cells, are essential to this process [18]. But to keep the immune system from attacking normal, healthy cells, it has “checkpoints” that function as brakes [18]. These are T-cell surface proteins, and they must be activated (or inactivated) to initiate an immunological response [18]. These checkpoints can be used by cancer cells to deter immune system attacks [19]. They possess the ability to express proteins that interact with these checkpoints and essentially shut down T cells, thus permitting the malignancy to spread unrestrained [19].

Checkpoint inhibitors have been approved for the treatment of a wide range of malignancies, particularly those that have proven to be resistant to conventional therapies like radiation or chemotherapy [20]. They are categorized based on the target molecule they each have, and they were first licensed for distinct malignancies in the European Union (EU) and the United States of America (USA) at different points in time, just as Table 1 describes below, but later on, it was discovered that they were also effective in other locations [20].

Numerous new immune checkpoint targets have been discovered recently, including VISTA, TIM-3, LAG-3, and T-cell Ig and immunoreceptor tyrosine-based inhibitory motif (ITIM) domain (TIGIT) [32]. These studies suggested that inhibiting one immunological checkpoint could cause the TME to upregulate other checkpoint receptors in a compensatory manner [33]. Lung cancer was found to have a comparable compensatory mechanism between TIM-3 and PD-1 [34].

One of the earliest and most successful checkpoint inhibitor applications was in melanoma. ICIs like Pembrolizumab, Nivolumab, and Ipilimumab have significantly improved survival rates for patients with advanced melanoma [22,29].

Regarding other cutaneous malignancies, ICIs such as Avelumab and Pembrolizumab are used to treat Merkel cell carcinoma, a rare and aggressive skin cancer, particularly in advanced stages, while Cemiplimab is used in the treatment of cutaneous CSCC [23,28,35].

ICIs like Pembrolizumab, Nivolumab, Atezolizumab, or Durvalumab are also widely used in advanced NSCLC, either as a first-line treatment or after chemotherapy [36,37]. Some patients are tested for PD-L1 expression to determine the likelihood of response to treatment. Atezolizumab and Durvalumab are often used in combination with chemotherapy as a first-line treatment for extensive-stage small cell lung cancer (SCLC) [38].

In RCC, ICIs like Nivolumab, Pembrolizumab, and Atezolizumab are used both alone and in combination with other therapies like tyrosine kinase inhibitors (e.g., Axitinib) to treat advanced kidney cancer [39].

ICIs like Atezolizumab, Pembrolizumab, Nivolumab, Durvalumab, and Avelumab are often used in patients with advanced or metastatic bladder cancer (urothelial carcinoma) who are not eligible for chemotherapy or after chemotherapy has failed [39].

Another use for ICIs such as Nivolumab, Pembrolizumab, and Atezolizumab (in combination with Bevacizumab) is advanced HCC, especially when other treatments like surgery or local therapies are not an option [40].

Pembrolizumab and Atezolizumab are ICIs that are used in combination with chemotherapy for advanced triple-negative breast cancer (TNBC), especially in patients whose tumors express PD-L1 [41].

Pembrolizumab and Nivolumab are two ICIs particularly effective in patients with microsatellite instability-high (MSI-H) or dMMR tumors, which are subtypes of CRC [42]. They are also effective in advanced or metastatic gastric and esophageal cancers, often in patients whose tumors express PD-L1 [43].

Pembrolizumab is also approved for use in advanced or metastatic cervical cancer with PD-L1 expression [44]. Pembrolizumab and Dostarlimab are typically used in advanced or recurrent cases of endometrial cancer, particularly for tumors with MSI-H or dMMR [45].

ICIs can be used in lymphatic malignancies other than solid tumors [46]. In primary mediastinal large B-cell lymphoma (PMBCL), Pembrolizumab is approved for use in this rare type of non-Hodgkin lymphoma, especially after other treatments have failed [47]. In Hodgkin lymphoma, ICIs like Pembrolizumab and Nivolumab have been highly effective in treating it, particularly in cases where the disease has relapsed or is resistant to other treatments [46].

Research indicates that progression-free survival and duration of response to Nivolumab (an anti-PD-1 antibody), Ipilimumab (an anti-CTLA-4 antibody), or Pembrolizumab (another anti-PD-1 antibody) are greater in individuals with high tumor mutational burdens (TMBs) compared to those with lower mutational burdens [48].

ICIs can interact with tumor cells and have antitumoral effects through a variety of mechanisms, such as the following:

### 2.1. Blocking PD-1/PD-L1 Interaction

PD-1 is a receptor on T cells, and PD-L1 is its ligand, which can be expressed in cancer cells [49,50,51]. When PD-1 on T cells binds to PD-L1 on cancer cells, it sends an inhibitory signal that reduces the T cell’s ability to kill the cancer cell [49,50,51]. This is a way for cancer cells to “turn off” the immune response [49,50,51]. Drugs like Pembrolizumab, Nivolumab, and Atezolizumab work by blocking this interaction. This blockage prevents the inhibitory signal from being sent, thereby allowing T cells to remain active and attack the cancer cells [49,50,51].

**Figure 1:**
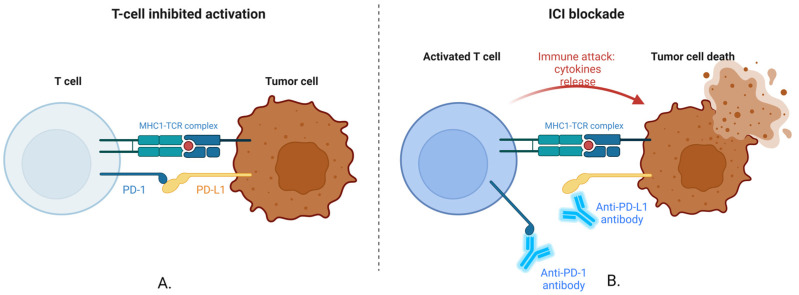
Inhibition of PD-L1/PD-1 checkpoint. (**A**)**. T-cell-Inhibited Activation.** T Cell: This immune cell is essential for locating and eliminating cancer cells. Tumor Cell: A malignant cell that uses inhibitory pathways to avoid detection by the immune system. MHC-TCR Complex: The tumor cell’s major histocompatibility complex (MHC) binds an antigen to the TCR. For T cells to identify the tumor cell, this contact is necessary. When activated, a T-cell receptor known as PD-1 transmits an inhibitory signal that lessens the T cell’s capacity to assault the tumor. PD-L1 is a ligand expressed on the surface of tumor cells and attaches itself to T-cell PD-1. By blocking T-cell activation, this interaction enables the tumor cell to avoid immune recognition and elimination. Since the tumor cell, in this instance, expresses PD-L1, which binds to the T cell’s PD-1 receptor, the tumor cell’s capacity to attack the T cell is hindered. The T cell receives a “stop” signal from this contact, which stops it from destroying the tumor cell. (**B**)**. ICI Blockade.** T cells that have been fully activated can mount an immunological response. Anti-PD-1/PD-L1 Antibodies: These antibodies prevent the interaction between the tumor cell’s PD-L1 and the T cell’s PD-1. The inhibitory signal is stopped by obstructing this contact, which keeps the T cell activated. Tumor Cell Death and Immune Attack: As a result of the antibodies blocking the inhibitory signals, the T cell releases cytokines and other cytotoxic chemicals, which ultimately cause the tumor cell to be destroyed. Anti-PD-1 or anti-PD-L1 antibodies, which are ICIs, in this case, block the interaction that would typically inhibit the T cell. As a result, the tumor cell is successfully attacked and killed by the T cell, which still functions. Created with BioRender.com (accessed on 18 August 2024).

The way ICIs strengthen the body’s defenses against cancer is depicted in Figure 1.

Providing a “brake” on the immune system, the PD-1/PD-L1 relationship normally stops T cells from attacking cells—including cancer cells—too aggressively [49,50,51]. To evade immune destruction, many tumors do, however, take advantage of this mechanism [49,50,51]. The immune system’s “brakes” are released by employing antibodies to obstruct this connection, which enables T cells to target and eliminate cancer cells more successfully [49,50,51]. The foundation of contemporary IT for several malignancies is this mechanism [49,50,51].

### 2.2. Blocking CTLA-4 Interaction

**Figure 2:**
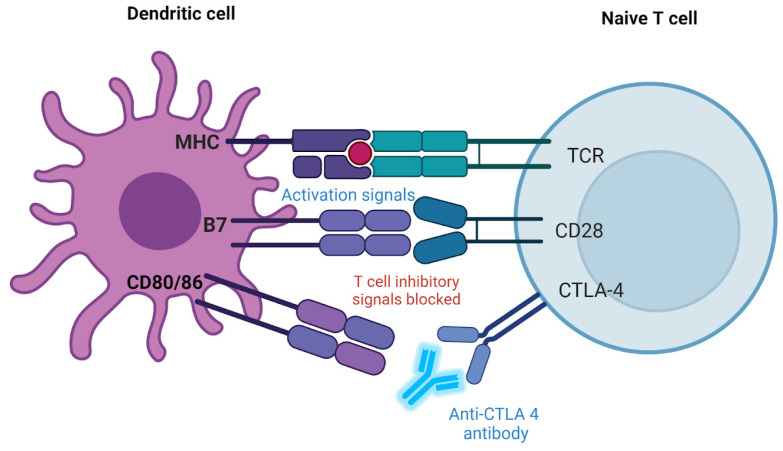
CTLA-4 checkpoint inhibition mechanism. Dendritic cell (DC): The APC type plays a crucial role in activating naive T cells. MHC: The MHC on the DC presents an antigen to the TCR on the naive T cell. This interaction is essential for T-cell activation. B7 molecules (CD80/CD86): These molecules on the DC surface bind to receptors on the T cell to either provide activation signals or engage in inhibitory signaling. Naive T cell: This T cell has not yet encountered its specific antigen. TCR: This receptor recognizes the antigen the MHC presents on the DC, initiating activation. CD28: A costimulatory receptor on the T cell that binds to B7 molecules (CD80/CD86) on the DC. When CD28 binds to B7, it provides a necessary costimulatory signal for T-cell activation, enhancing its response. CTLA-4: This receptor is also on the T cell and competes with CD28 for binding to B7. However, when CTLA-4 binds to B7, it sends an inhibitory signal, dampening the T cell’s activation to prevent excessive immune responses. Anti-CTLA-4 antibody: The figure shows an anti-CTLA-4 antibody blocking the CTLA-4 receptor. This prevents CTLA-4 from binding to B7 molecules, thereby blocking the inhibitory signal normally downregulating T-cell activation. By blocking CTLA-4, the antibody enhances the activation signal from the CD28-B7 interaction, promoting a stronger immune response. Created with BioRender.com (accessed on 10 August 2024).

CTLA-4 is another inhibitory receptor found on T cells. It competes with the stimulatory receptor CD28 to bind to B7 molecules (B7-1/CD80 and B7-2/CD86) on APCs [49,50,51]. When CTLA-4 binds to B7, it sends an inhibitory signal to the T-cell, dampening the immune response. Drugs like Ipilimumab block CTLA-4, which prevents it from binding to B7 molecules [49,50,51]. This blockage allows CD28 to bind to B7 instead, promoting T-cell activation and enhancing the immune response against cancer cells [49,50,51]. Figure 2 shows how an anti-CTLA-4 antibody can control the ratio of T-cell activation to inhibition.

Typically, CTLA-4 acts as a “checkpoint” to prevent T-cell activation and limit the immune system’s overreaction. Nonetheless, using an antibody to suppress CTLA-4 can strengthen and prolong T-cell activation in cancer treatment, boosting the immune response against tumor cells [51]. Certain immunotherapies used to treat cancer are based on this process [29,30].

### 2.3. Enhanced T-Cell Activation and Proliferation

T cells, when not inhibited by checkpoint pathways, can proliferate more effectively and exert stronger antitumor activity [52]. The immune response is more robust, with a higher number of active T cells available to target and destroy cancer cells [52]. By blocking the inhibitory signals (like PD-1/PD-L1 or CTLA-4), checkpoint inhibitors enhance the activation and proliferation of T cells. This leads to a more powerful immune response against the tumor [52].

### 2.4. Induction of Immunological Memory

A successful immune response against cancer can lead to the generation of memory T cells, which can remember and respond more quickly to cancer cells if they return [53]. Because checkpoint inhibitors can enhance the initial immune response, they may also promote the development of immunological memory [51]. This means the immune system can remain vigilant and potentially prevent cancer recurrence [51].

### 2.5. Reactivation of Exhausted T Cells

In the tumor microenvironment, T cells can become “exhausted” due to chronic exposure to cancer antigens and continuous inhibitory signaling [54]. Exhausted T cells are less effective at attacking cancer cells [54]. Checkpoint inhibitors can rejuvenate these exhausted T cells by blocking the inhibitory signals (such as PD-1/PD-L1), restoring their function and enabling them to resume their attack on cancer cells [55].

### 2.6. Overcoming Tumor-Induced Immunosuppression

Tumors can create an immunosuppressive environment by secreting various factors such as transforming growth factor beta (TGF-β) and interleukin 10 (IL-10) or by recruiting regulatory cells (like regulatory T cells and myeloid-derived suppressor cells (MDSCs) that inhibit T-cell function [56]. While not directly targeting these factors, checkpoint inhibitors can help overcome some of the tumor-induced immunosuppression by reinvigorating T cells and reducing the influence of the tumor’s immunosuppressive strategies [57,58].

### 2.7. Synergy with Other Immune Cells

Besides T cells, other immune cells like DCs (which present antigens) and natural killer (NK) cells also play roles in antitumor immunity [59]. By enhancing T-cell function, checkpoint inhibitors may also indirectly improve the activity of other immune cells, leading to a more coordinated and effective immune attack on the tumor [57].

ICIs, which allow the immune system to identify and target tumor cells, have completely changed the treatment of cancer [50]. But not every patient responds to these treatments, and resistance may arise from internal (primary) or external sources (secondary) [1]. There are several ways in which tumors may resist the effects of ICIs [1].

Several factors, including lack of tumor antigen presentation, low TMB, absence of infiltrating T cells (“Cold tumors”), inhibitory immune checkpoints beyond PD-1/PD-L1 and CTLA-4, or immunosuppressive cytokines, can lead to intrinsic (primary) resistance, which exists before the start of treatment and prevents the therapy from being effective at all [60,61].

- Lack of tumor antigen presentation: Cancer cells must present antigens via the MHC for T lymphocytes to identify and destroy them [62]. T cells become resistant when tumors downregulate or stop expressing MHC molecules, which prevents the T cells from identifying the cancer cells [62].
- TMB: Neoantigens, or novel antigens that the immune system can target, are produced in smaller quantities by tumors with fewer mutations [63,64]. ICIs are less effective when the TMB is low because the immune system has fewer targets [63,64].
- Absence of infiltrating T cells (“Cold tumors”): Often called “cold” tumors, some cancers have an immunosuppressive microenvironment that hinders T cells from penetrating the tumor [65]. ICIs are ineffective without T-cell infiltration [65].
- Inhibitory immune checkpoints beyond PD-1/PD-L1 and CTLA-4: Other immune checkpoint molecules that can similarly suppress T-cell function, such as TIM-3, LAG-3, or VISTA, may also be expressed by tumors [66,67]. If these other pathways are activated, it might not be enough to use ICIs that only target CTLA-4 or PD-1/PD-L1 [66,67].
- Immunosuppressive cytokines: Certain tumors release cytokines, such as vascular endothelial growth factor (VEGF), IL-10, or TGF-β, that impair the immune system and produce an environment that is unfavorable to T-cell activation [56,68,69].
- After a first response to the therapy, acquired (secondary) resistance arises, which ultimately results in treatment failure [70]. It can arise from a variety of mechanisms, including signaling pathway mutations, decreased neoantigen expression, upregulation of alternative immune checkpoints, elevated PD-L1 expression, T-cell exhaustion, metabolic changes in the tumor microenvironment, or immunosuppressive cell recruitment [71].
- Mutations in signaling pathways: Signaling pathway abnormalities in tumor cells can enable them to evade immune recognition [72,73,74]. A tumor’s capacity to react to interferon-gamma (IFN-γ), a cytokine essential for immune-mediated tumor elimination, can be hindered by mutations in the JAK1/2 gene, for instance [72,73,74].
- Loss of neoantigen expression: Neoantigens that were first identified by the immune system may disappear from tumors through evolution, preventing T cells from identifying and eliminating them [75,76].
- Upregulation of alternative immune checkpoints: Tumors may upregulate additional inhibitory molecules such as TIM-3, LAG-3, or VISTA after PD-1/PD-L1 or CTLA-4 pathways are inhibited, offering other possibilities to suppress the immune response [77,78].
- Increased expression of PD-L1: In reaction to the immune attack, tumor cells might adapt by upregulating their expression of PD-L1, which can overcome the blockage offered by anti-PD-1/PD-L1 antibodies [79,80].
- T-cell exhaustion: The process in which T cells lose their capacity to operate normally, even in the presence of ICIs, might result from prolonged contact with the tumor environment. Tired T cells do not make enough cytokines and display a lot of inhibitory receptors [81,82].
- Metabolic alterations in the tumor microenvironment: Tumors can produce metabolic wastes like lactic acid that inhibit immune cell activity or alter their microenvironment to deprive immune cells of vital resources like glucose and amino acids [83,84].
- Recruitment of immunosuppressive cells: The action of ICIs can be countered by tumors attracting regulatory T cells (Tregs), MDSCs, and tumor-associated macrophages (TAMs), which together generate an immunosuppressive environment [85,86].

The TME influences acquired and inherent mechanisms, which is vital in resistance [87]. As previously noted, the TME contains Tregs, MDSCs, and TAMs that can suppress effector T-cell activity and neutralize the effects of ICIs [88]. Due to the creation of barriers to immune cell infiltration and the promotion of molecules like VEGF production, hypoxia within the tumor might result in an immunosuppressive environment [89,90]. ICI efficacy may be restricted by dense stromal tissue in the TME, which may physically prevent immune cells from penetrating the tumor [91,92].

When it comes to ICIs, there are various ways to overcome resistance [1]. One such tactic is to combine ICIs with other therapies like radiation, chemotherapy, targeted therapy, or other immune-modulating medicines. This way, the tumor will be attacked from multiple angles, helping to overcome resistance [93,94]. To overcome resistance to CTLA-4 or PD-1/PD-L1 inhibitors, drugs targeting other immune checkpoints such as LAG-3, TIM-3, or TIGIT are also being studied [95]. Conversely, treatments that alter the TME to promote immune cell activity—such as those that target MDSCs or Tregs, lower hypoxia, or weaken stromal barriers—can aid in overcoming resistance [95].

In conclusion, the interaction between checkpoint inhibitors and cancer cells is primarily focused on removing the inhibitory signals that prevent the immune system from attacking the cancer [96]. By blocking proteins like PD-1, PD-L1, and CTLA-4, these drugs enable a stronger and more sustained immune response, leading to the destruction of cancer cells [97]. This process involves multiple mechanisms, including direct T-cell activation, overcoming immunosuppression, and potentially inducing long-term immunological memory [56]. Despite the great potential of ICIs, resistance remains a significant issue. Understanding the reasons causing this resistance is crucial to developing strategies to improve the efficacy of these medicines.

## 3. Exploratory Biomarkers for Immune Checkpoint Inhibition

As discussed in the next subchapter, several predictive biomarkers have already received FDA approval and are utilized to monitor the therapeutic response to ICIs. Recent research is concentrating on evaluating the effectiveness of new biomarkers, including DNA damage response (DDR) gene alterations, MHC genotypes, beta-2-microglobulin (B2M) deficiency, polymerase epsilon (POLE) mutations, Janus kinase 1/2 (JAK1/2) mutations, tumor-infiltrating T cells (TILs), peripheral T cells, and the gut microbiome [98]. The latter will be discussed separately in a separate section of this article.

The DDR represents the cellular response to both endogenous and exogenous DNA damage, being implicated in repair processes through different pathways: dMMR, direct reversal, homologous recombination repair, nucleotide excision repair, base excision repair, and non-homologous end joining [98,99,100]. Recent studies have shown that immune checkpoint blockade holds great promise for NSCLCs. In a study by Chae et al., the authors examined a predefined list of 812 immune metagenes, derived from 813 microarrays across 36 studies, to accurately predict the presence of up to 28 intratumoral immune cell types in a large cohort of lung adenocarcinoma patients from The Cancer Genome Atlas. It was demonstrated that tumors with DNA repair deficiencies (n = 230), particularly in the dMMR pathway, which can accelerate mutation rates and impact prognosis, have significant potential to be used as biomarkers for TMB, neoantigen load, and tumor-infiltrating lymphocytes in patients undergoing anti-PD-1/PD-L1 therapy [98,101]. In urothelial cancer (UC), recent studies have indicated reduced response rates to IT, yet still an improvement overall compared to previous systemic therapies. In a study by Teo et al., the researchers hypothesized that DDR gene mutations could serve as biomarkers for anti-PD-1/PD-L1 therapy in patients with metastatic UC [102]. They analyzed sixty patients with metastatic UC treated with anti-PD-1/PD-L1 antibodies, enrolled in three separate prospective trials between April 2014 and December 2016, and identified seventy-four DDR gene alterations, including twenty-seven deleterious DDR gene alterations, before treatment initiation [102]. Patients with deleterious DDR gene alterations exhibited a lower incidence of hepatic involvement and were associated with a higher TMB [102]. Their findings showed that DDR gene alterations were linked to clinical benefits in patients receiving anti-PD-1/PD-L1 therapy, with more pronounced effects observed in those with deleterious DDR alterations (80%) and in those whose tumors had wild-type DDR genes (19%; *p* = 0.001) [102]. Additionally, Teo et al. emphasized the significance of deleterious DDR alterations beyond MMR deficiency, which constituted only 5% of their study cohort, in contrast to findings by Chae et al. [101,102].

MHC-I molecules present intracellular proteins to T cells, enabling them to identify mutated or foreign peptides, which influences antigenicity [98]. Recent research indicates that the insufficient presentation of driver mutation neoantigens by MHC-I may explain why certain tumors do not respond to ICIs [98]. MHC-II molecules, found specifically in APCs such as macrophages and DCs (DC), have also been studied. Recent findings suggest that human leukocyte antigen-DR isotype (HLA-DR), a component of MHC-II, is elevated in malignant cells and is associated with favorable prognosis and improved response to ICI treatment [103,104]. In a study conducted by Johnson et al., the authors analyzed two independent cohorts of melanoma patients treated with anti-PD-1 therapy, and they highlighted that malignant cells, particularly melanoma cells, with high levels of HLA-DR are more responsive to PD-1 antibodies due to their inflammatory signals, even in patients with poor prognosis, such as those with liver metastases [105]. In their study, 75% of HLA-DR-positive patients responded to anti-PD-1 therapy, compared to only 27% of HLA-DR-negative patients (*p* = 0.025), and they also demonstrated a significantly better survival rate (median not reached versus 27.5 months, log-rank *p* = 0.003) [104]. Additionally, a study by Jiang et al. evaluated the IT response in 85 NSCLC patients, using HLA typing, revealing that those expressing HLA-DRB4 had an improved overall survival of 26.3 months compared to 10.2 months in untreated patients. This finding was significant in both univariate (*p* = 0.022; 26.3 months vs. 10.2 months) and multivariate analysis (*p* = 0.011, HR 0.49, 95% CI = 0.29–0.85), emphasizing the significance of MHC-II in the antitumor immune response during ICI treatment [104]. However, Jiang et al. also noted an increased risk of endocrine adverse reactions in patients with HLA-DRB4 undergoing ICI treatment (11 patients, 81.8% had HLA-DRB4, *p* = 0.0139), although the cause remains unknown [104]. Importantly, Jiang et al. found that patients who developed adverse reactions to ICIs had increased overall survival rates, underscoring the complex relationship between treatment response and adverse reactions [104].

Recent studies have shown that B2M deficiency, a subunit of MHC-1, can downregulate MHC-1 antigen presentation, thereby increasing cancer immune evasion [98]. In a study by Sade-Feldman, the authors analyzed longitudinal tumor biopsies from 17 metastatic melanoma patients and observed that B2M deficiency was more prevalent in non-responder patients (29.4%) compared to responders (10%) treated with anti-CTLA-4 or anti-PD-1 therapy. This deficiency was associated with poorer survival rates, suggesting that B2M could be a potential biomarker for therapy resistance [106]. The study highlighted that one of the main reasons for this resistance is the absence of proteins that can substitute for B2M in HLA class I presentation [106].

POLE is a crucial eukaryotic DNA polymerase involved in DNA replication and repair. A study by Rousseau et al. discovered that POLE mutations can serve as predictive biomarkers for patients with CRC or endometrial carcinoma treated with PD-1 antibodies, as these mutations are associated with increased TMB and tumor-infiltrating lymphocytes [107]. The study highlighted that POLE mutations are primarily found in young male patients with CRC and young female patients with endometrial carcinoma, often indicating a favorable prognosis [107]. Their analysis revealed that tumors harboring POLE mutations exhibited higher levels of cytotoxic T helper (Th) 1 cell infiltrates and PD-1 expression, along with significant upregulation of IFN-γ, PD-L1, and CTLA-4, which are critical for patients undergoing ICI therapy [107]. Using Nivolumab, an anti-PD-1 antibody therapy, the study observed therapeutic responses exclusively in CRC and endometrial carcinoma patients with POLE mutations, reporting a survival rate of 5.4 months [107]. Remarkably, they noted a near-complete response in a 42-year-old patient with unresectable CRC and POLE mutations [107]. The study also emphasized the correlation between dMMR and POLE mutations, suggesting that DNA repair defects can be effective biomarkers for ICI treatment in certain cancer types [107]. Furthermore, they underscored the importance of associating POLE mutations with low-TMB tumors, which do not benefit from PD-1 blockade, in contrast to high-TMB tumors [107].

JAK1/2-inactivating mutations have been shown to increase tumor resistance to Pembrolizumab, an anti-PD-1 therapy, by disabling the IFN-γ responsive pathway [98]. In a study by Shin et al., these mutations were analyzed in patients with melanoma or CRC and high TMB that did not respond to anti-PD-1 therapy due to the diminished IFN-γ pathway and consequent lack of PD-L1 expression [108]. This finding is intriguing, as many studies have shown that patients with high TMB generally respond better to IT [91]. Biopsies from non-responder patients with advanced melanoma and high TMB revealed JAK1 domain mutations, along with low or absent CD8 infiltrates and PD-1/PD-L1 expression [108]. Conversely, in responder patients, JAK2 mutations were observed but lacked the same functional impact [108]. The results indicated that patients with JAK1 mutations did not respond to IFN-α, IFN-β, or IFN-γ, and consequently to anti-PD-1 therapy, whereas patients with JAK2 mutations were unresponsive only to IFN-γ but showed PD-L1 upregulation when exposed to IFN-α or IFN-β [108]. Nonetheless, survival rates for patients with tumors harboring JAK1/2 mutations were significantly lower [108]. The study concluded that JAK1/2-inactivating mutations are both acquired and primary causes of resistance to anti-PD-1 therapy, serving as effective predictive markers for this resistance [108].

TILs, especially tumor-reactive cytotoxic CD8+ T cells, have been linked to better responses in patients with various tumor types treated with ICIs [98,109,110]. Tissue-resident memory T cells (TRM), a recently discovered subtype of CD8+ memory T cells, have shown promise in improving outcomes for patients with NSCLC, oral, or breast cancer treated with anti-PD-1 therapy [98,109,110]. These cells enhance responses by blocking PD-1 or CTLA-4 expression, making them potential biomarkers for ICI treatment [98,109,110].

Corgnac et al. conducted a retrospective study on a cohort of 111 patients with NSCLC, discovering that those with higher pre-IT levels of CD8+ TRM cells, characterized by the CD103+CD49a+CD69+ phenotype, had increased TRM levels during anti-PD-1 treatment. This was associated with improved outcomes compared to non-responders who had low CD8+ levels (HR = 0.40, 95% confidence interval [CI], 0.20–0.77, *p* = 0.006). The median immunotherapy progression-free survival for these patients was 30 months (95% CI, 2.73, not reached) [109]. This improvement was attributed to the higher presence of activated tumor antigen-reactive T cells. Thus, TRM cells were suggested as potential biomarkers for ICI treatment in NSCLC patients [109].

Another study by Lee et al. demonstrated that TRM cells with the CD39+ phenotype contributed to favorable responses in patients with triple-negative breast cancer treated with ICIs and predicted patient survival, primarily due to their functional restoration capability [110]. Importantly, CD39+ TRM cells influenced both local and systemic antitumor immunity. Their study also showed that combining anti-CTLA-4 and anti-PD-1 therapy yielded better outcomes than anti-PD-1 therapy alone in these patients [110].

Chronic antigen stimulation leads to the exhaustion of tumor-reactive cytotoxic CD8+ T cells, giving rise to exhausted T cells and their precursors (TPEX) [111]. These TPEX cells have been recently studied in melanoma and NSCLC patients undergoing anti-PD-1 therapy, being correlated with enhanced response duration, suggesting their potential as biomarkers for ICI treatment [98,111].

Miller et al. found that TPEX cells were more effective than terminally exhausted T cells in controlling tumor growth [111]. In their study of melanoma patients who responded to ICI therapy, they observed higher expression of TPEX cells compared to terminally exhausted T cells, concluding that TPEX cells could be biomarkers for longer response duration to ICIs in melanoma patients [111]. Similarly, Liu et al. observed higher TPEX cell levels and lower terminally exhausted T-cell levels in NSCLC responders compared to non-responders following anti-PD-1 therapy, reinforcing the potential of TPEX cells as biomarkers [112].

Immuno-oncology has undergone significant changes over time due to rapid advancements in research. Today, thousands of potential therapies are available in immuno-oncology, thanks to robust drug development pipelines. However, despite promising preclinical data, some studies have observed hyperprogressive disease in patients during later clinical trials when treated with a combination of immune checkpoint inhibitors (ICIs) and other immuno-oncology therapies [48].

Chen et al. conducted a comprehensive pan-cancer analysis, examining the prevalence of Kelch-like ECH-associated protein 1 (KEAP1) mutations in a cohort of 40,167 patients with various cancer types (including lung cancer, endometrial cancer, hepatocellular carcinoma, head and neck cancer, bladder cancer, colorectal cancer, and esophagogastric cancer) using data from the cBioPortal online database [113]. They found that the overall prevalence of KEAP1 mutations was 2.7%, with the highest prevalence observed in patients with NSCLC (15.8%) [113]. The study also revealed that patients with KEAP1 missense mutations exhibited the highest TMB levels, indicating that KEAP1 mutations could serve as negative predictive biomarkers for immunotherapy outcomes [113]. The authors demonstrated the prognostic significance of KEAP1 mutations in both early-stage (*p* = 0.0099) and advanced-stage cancers (*p* < 0.0001) [113]. Moreover, when correlating KEAP1 mutations with overall survival, they found that patients with these mutations had a significantly reduced overall survival rate (39 months) compared to those without KEAP1 mutations (109 months) (*p* < 0.0001) and shorter disease-free survival (97 vs. 158 months, *p* = 0.0677) [113]. A contributing factor to these outcomes was the significantly lower infiltration of CD8+ T cells in patients with KEAP1 mutations, particularly in endometrial cancer, breast cancer, bladder cancer, colorectal cancer, lung adenocarcinoma, and lung squamous cell carcinoma, with the lowest levels observed in lung adenocarcinoma [113].

In a retrospective study by Papillon-Cavanagh et al., the researchers analyzed mutations in KEAP1 and serine/threonine kinase 11 (STK11) among 2276 patients with stage IIIB, IIIC, IVA, or IVB NCSLC, including 574 patients who received anti-PD-1/anti-PD-L1 inhibitor treatment. The study found that both STK11 and KEAP1 mutations were associated with a poor response across multiple therapeutic classes [114]. However, this poor response was not specifically observed in the cohort treated with ICIs, nor was there a significant impact on progression-free survival or overall survival. For STK11 mutations, the hazard ratios were HR 1.05 (95% CI 0.76 to 1.44, *p* = 0.785) for progression-free survival and HR 1.13 (95% CI 0.76 to 1.67, *p* = 0.540) for overall survival [114]. For KEAP1 mutations, the hazard ratios were HR 0.93 (95% CI 0.67 to 1.28, *p* = 0.653) for progression-free survival and HR 0.98 (95% CI 0.66 to 1.45, *p* = 0.913) for overall survival [114]. The authors concluded that these mutations serve more as prognostic biomarkers rather than predictive ones for anti-PD-1/anti-PD-L1 therapy [114].

In contrast, many studies have highlighted the poor prognosis of patients with STK11 gene codes for liver kinase B1 (STK11/LKB1) and KEAP1 mutations treated with ICIs. However, recent research evaluating their impact in KRAS wild-type patients, or independent of KRAS mutations, suggests that the poor prognosis is primarily linked to concurrent KRAS mutations [115]. These contradictory findings largely stem from study limitations, such as the inclusion of other gene mutations and the lack of comparison arms involving patients who received alternative treatments [115].

Recent studies have emphasized the significance of targeting the nuclear factor erythroid 2-related factor 2 (NRF2)–KEAP1 axis in various diseases, including cancer, due to the cytoprotective functions of proteins encoded by NRF2-target genes, such as those involved in antioxidant defense, detoxification, and anti-inflammatory processes [116]. However, targeting the NRF2-KEAP1 axis was not previously considered a priority, largely because of its extensive range of biochemical activities that extend beyond the regulation of antioxidant systems, many of which are governed either directly or indirectly by NRF2, leading to nonspecific effects [116]. The benefits of targeting the NRF2-KEAP1 axis have only recently gained attention, including the maintenance of redox signaling, enhanced biotransformation of xenobiotics, regulation and resolution of inflammation, suppression of gluconeogenesis and hepatic lipogenesis, support of proteostasis, and inhibition of fibrosis [116]. Recent research has identified numerous effective drugs targeting the NRF2-KEAP1 axis for both neoplastic and non-neoplastic diseases, with many already approved and in use, and several others still under investigation [116]. Table 2 summarizes drugs targeting the NRF2-KEAP1 axis that have shown efficacy in certain types of cancer.

In a comprehensive pan-cancer analysis by Lee et al., researchers examined the genomic landscape of Kirsten rat sarcoma viral oncogene homolog (KRAS)-altered solid and hematologic malignancies in 426,706 patients treated with chemotherapy, chemotherapy combined with ICIs, or ICI monotherapy [119]. They found that KRAS alterations were present in 23% of the cases, with 88% being mutations, most commonly observed in CRC with KRAS G12D and G12V mutations, followed by NSCLC with KRAS G12C mutations [119]. The study also evaluated co-altered genes related to immunotherapy response, including TMB and PD-L1 expression, as well as co-mutations in STK11 and KEAP1, which were associated with low (28% and 16%) or negative (53% and 27%) PD-L1 expression, respectively [119]. The researchers observed similar overall survival rates in NSCLC patients with KRAS G12C and KRAS G12V mutations (11 vs. 10 months, HR 1.0, 95% CI 0.86–1.20, *p* = 0.88) and KRAS G12D mutations (11 vs. 12 months, HR 0.91, 95% CI 0.75–1.11, *p* = 0.36) [119]. Notably, patients with KRAS alterations had decreased overall survival compared to those without KRAS alterations but with other oncogenic mutations (11 vs. 27 months, HR 0.55, 95% CI 0.49–0.63, *p* < 0.001) [119]. Similar findings were reported in CRC and other cancer types, emphasizing the significant implications for developing KRAS inhibitors, particularly targeting G12C, G12D, and G12V mutations [119]. The authors acknowledged study limitations, including the lack of detailed clinical and treatment information for patients in the database and the selection bias introduced by using comprehensive genomic profiling [119].

Selective KRAS G12C inhibitors are the only treatments that have shown clinical benefits in patients with NSCLC and KRAS G12C mutations who have undergone at least one prior systemic therapy [117]. Sotorasib, an approved selective KRAS G12C inhibitor, has demonstrated clinical efficacy and a tolerable safety profile in these patients [117]. Similarly, Adagrasib, another approved KRAS G12C inhibitor, has proven effective in pretreated NSCLC patients [117]. However, some newer inhibitors are still under investigation and face limitations due to intrinsic and acquired resistance mechanisms. KRAS mutations are also present in pancreatic ductal adenocarcinoma and CRC [117].

Sotorasib is a KRAS G12C inhibitor that binds irreversibly to the S-IIP pocket, locking KRAS in its inactive GDP-bound state and inhibiting KRAS oncogenic signaling [117]. Additionally, Sotorasib enhances its interaction with KRAS G12C and increases potency by binding to a surface groove created by an alternative orientation of histidine at position 95, surpassing the effectiveness of AMG-1620, another KRAS G12C inhibitor [117]. Studies using xenograft models have demonstrated that Sotorasib can inhibit ERK phosphorylation and tumor cell growth in KRAS G12C-mutant cells, leading to durable tumor regression as a monotherapy [117]. It can also be used in combination with chemotherapy [117].

Adagrasib is another KRAS G12C inhibitor with a mechanism similar to that of Sotorasib, irreversibly binding to the mutant protein and locking it in its inactive GDP-bound form [117]. However, Adagrasib offers some advantages over Sotorasib, including high oral bioavailability, a long half-life (approximately 24 h), extensive tissue distribution, and the ability to penetrate the central nervous system [117]. Notably, studies have shown that Adagrasib exhibits synergistic effects when combined with inhibitors of the EGFR family, SHP2, mTOR, or CDK4/6 [117]. Table 2 provides a summary of the key drugs used to inhibit the KRAS G12C mutation across various cancer types.

Additionally, other mutations, such as mesenchymal–epithelial transition (MET), human epidermal growth factor receptor 2 (HER2), rearranged during transfection (RET), and neurotrophic tyrosine receptor kinase (NTRK), are still under study and have shown promising clinical potential for the development of specific inhibitors [117].

## 4. Approved Biomarkers for Immune Checkpoint Inhibition

Despite the remarkable efficacy of ICIs, many patients (~70%) do not respond well or develop resistance to these drugs [120]. The response rate to ICIs varies between 15% and 30% in most solid tumors and 45% and 60% in MSI-H cancers and malignant melanoma [121].

Currently, the FDA has only approved three predictive biomarkers for ICI therapy in malignancies [122]. These biomarkers are PD-L1, MSI or dMMR, and TMB [122].

PD-L1, also known as programmed death ligand 1, has gained recognition as a significant biomarker in breast cancer in recent times [123]. There is a correlation between this factor and poorer outcomes in patients with hormone receptor-positive breast cancer. However, it has a predictive function in guiding the response to systemic treatment specifically in the TNBC subtype, particularly in cases where the cancer has spread [123]. ICIs are now being incorporated into the treatment regimen for a growing number of patients with TNBC [123].

Monoclonal antibody IT that targets PD-1 andvPD-L1 has become a widely accepted treatment for patients with lung cancer (NSCLC) [124]. It is used in both initial and subsequent treatment stages, and has shown long-lasting positive responses in around 10-20% of treated patients [124].

In 1992, T. Honjo and his colleagues at Kyoto University discovered that PD-1 is a gene related to apoptosis [125]. However, the process of apoptosis does not necessitate the overexpression of PD-1 [125].

PD-1 function requires tyrosine in T and B lymphocytes [126]. The orderliness in the cytoplasmic tail of PD-1 can be bound by protein tyrosine phosphatase 1 and 2 (SHP-1, SHP-2) [126]. However, it is unknown whether PD-1 takes SHP-1 and/or SHP-2 in a physiological setting. Cysteine is responsible for the homodimerization of CD28, CTLA-4, and inducible costimulatory molecules [127]. On the other hand, PD-1 is monomeric on the cell surface and is unable to form covalent homodimers because it lacks the extracellular surface [128].

Subsequent research conducted in 1996 by the same group revealed that PD-1 expression was stimulated by antigen receptors on T and B cells [129] and had a role in suppressing immunological responses, since PD-1-deficient mice displayed autoimmune symptoms [130,131].

Nobel prize laureate Tasuku Honjo and his team have used J43 mAb immunoprecipitation and flow cytometry to determine the PD-1 gene product, which is a 50–55 kDa membrane protein produced on the cell surface of 2B4.11 cells, and various PD-1 cDNA transfectants [132].

The PD-1 antigen is present in specific subsets of thymocytes and spleen T cells, which may cause T-cell activation or death [129]. In a similar manner, in vitro treatment with an anti-Ig M antibody caused PD-1 expression on spleen B cells [129]. PD-1, on the other hand, was not substantially expressed on lymphocytes after treatment with lipopolysaccharide, dexamethasone, or growth factor deprivation [129]. The findings indicate that PD-1 antigen expression is closely controlled and stimulated by signal transduction via the antigen receptor [129]. While PD-1 expression is not necessary for the common process of apoptosis, it remains possible that the PD-1 antigen contributes to lymphocyte clonal selection [133].

PD-1 functions as PD-L1’s T-cell-inhibitory receptor, and both PD-L1 (CD274) and PD-L2 (CD273; also known as B7-DC) are expressed in cancer cells and APCs in the TME [134,135,136].

The goal of targeting PD-1 and its ligands is to attack tumors with the help of the body’s immune system [137,138]. The development and use of such blocking antibodies fundamentally altered the course of cancer IT [137,138].

PD-1 is activated in several peripheral hematopoietic cells by cytokines and antigen receptor signaling; its expression occurs throughout thymic development [127]. During TCR β rearrangement, immature CD4−CD8− (double negative) thymocytes express PD-1 [139]. Upon their activation, peripheral CD4+ and CD8+ T cells, B cells, monocytes, NK T cells, and certain subsets of DCs express PD-1 through induction [140].

PD-1 on T cells and APCs can be induced by estrogen [141]. As a result of being activated, peripheral T cells (CD4+ and CD8+), B cells, NK T cells, monocytes, and certain types of DCs (DC) show PD-1 expression. BCR or TCR activation makes PD-1 function [142].

Macrophages, memory B cells, and mast cells exhibit inducible PD-L2; PD-L2 is expressed in fewer cell types compared with PD-L1 [126,143]. Hematopoietic and non-hematopoietic cells generally express PD-L1 [144]. Although produced constitutively in T cells, PD-L1 levels are raised after the activation of B cells, DCs, macrophages, and mast cells originating from bone marrow [127].

Mice naturally express more PD-L1 than humans [127]. Neurons, pancreatic islet cells, endothelial cells, epithelial cells, fibroblastic reticular cells, and cells identified in immune-privileged regions, such as retinal pigment epithelial cells and the placenta, all contain PD-L1 [127].

Due to the widespread expression of PD-L1 on both hematological and non-hematopoietic cells, and the expression of PD-1 on B cells, T cells, some DCs, and macrophages, there are several potential interactions in both directions between PD-1 and PD-L1 [145]. T cells excel in facilitating the function of PD-1 [145].

PD-1 interacts with one of its ligands during TCR signaling, reducing T-cell survival and inhibiting T-cell proliferation, cytokine generation, and cytolytic activity [146,147].

PD-Ls have been shown in several studies to have the ability to inhibit T-cell cytokine production and cell proliferation. However, they have also been seen to stimulate T-cell activation. The origins of these divergent results are now unclear [148,149].

Immune cells that target particular antigens and do not have PD-1 receptors respond more strongly to antigen-containing resting DCs, mostly via CD8+ T cells. It is the antigen itself that causes this reaction [150]. A negative impact on effector T-cell reactivation, proliferation, and function may also be exerted via the PD-1/PD-L pathway [148]. Some tumor-penetrant cancer cells and APCs may also display PD-1/PD-L1 interaction in cis when these cells express PD-1 and PD-L1, according to recent studies [151]. This interaction is called cis expression [151]. Researchers found that when PD-1 and PD-L1 are produced on the same cancer or antigen-presenting cell (APC), they touch each other directly on the cell surface. Hence, this contact, called cis interaction, reduces the capacity of PD-L1 to bind to PD-1 on T cells in a trans manner [152,153]. This goal was achieved by the creation of new methods for re-creating lipid bilayer systems in vitro and by conducting experiments in cell cultures [154].

### 4.1. MSI or dMMR

Repetitive DNA sequences (repeats) are scattered across the human genomes, e.g., repeats of mononucleotides, dinucleotides, or higher-order nucleotide repeats like (A)n or (CA)n make microsatellites. Because polymerases slip during DNA synthesis, these sequence motifs are particularly vulnerable to the accumulation of mutations [155]. The MMR system is in charge of monitoring and fixing mistakes made in microsatellites [156]. Microsatellites, also known as short tandem repeats, are genome-wide repeating DNA sequences that are dispersed throughout both coding and noncoding areas [156]. Their unit lengths range from one (mononucleotides) to six bases (tetra-, penta-, esa-, and trinucleotides) [156]. Although they vary greatly throughout themes, they are constant in every single person [157,158]. MSI, which is easily detected by the study of polyA microsatellites, is the result of the accumulation of mutations resulting in repeat length variations in dMMR [157,158,159]. These areas are especially sensitive to mismatch errors because of their repeating nature. MSI thus indicates a hypermutable condition of cells and serves as a hallmark of dMMR [157,158,159].

Maintaining genomic integrity is largely dependent on the highly conserved biological system known as dMMR [160]. Base–base mismatches and insertion/deletion mispairs produced during DNA replication and recombination are the main sources of MMR’s specificity [160]. Additionally, MMR inhibits homologous recombination and has been implicated in the signaling of DNA damage in eukaryotic cells [160]. The primary errors associated with microsatellites are insertion–deletion loops, which refer to extrahelical nucleotides that form DNA hairpins, and base–base mismatches, which bypass the intrinsic proofreading mechanism of DNA polymerases [161]. When the starting nucleotide and template strand split and recombine incorrectly in a microsatellite, it leads to the formation of partnerless nucleotides [161]. Frameshift mutations that lead to protein truncations are generated by insertions or deletions in microsatellites located in coding regions of DNA [161].

When DNA replication, iatrogenic damage, or recombination leads to a mismatch, the process of dMMR comes into play to resolve the issue [160,162,163]. Four genes—MLH1, MSH2, MSH6, and PMS2—control the mitotic spindle region (MMR) process [160,162,163]. Two genes, MLH1/PMS2 and MSH2/MSH6, encode separate proteins [160,162,163]. The mismatch repair genes MLH1, MSH2, MSH6, and PMS2 have germline mutations or epigenetic silencing of MLH1 and account for approximately 15% of CRC with MSI [164]. The mismatch repair genes MLH1, MSH2, MSH6, and PMS2 have germline mutations or epigenetic silencing of MLH1, and account for approximately 15% of CRC with MSI [164].

The idea of an MSI phenotype in CRC originated from the description of the clinical and molecular characteristics of MSI tumors [165]. Research has shown that MSI tumors are more likely to respond favorably to chemotherapy than microsatellite-stable CRC; however, there may be differences in how MSI cancers react to these treatments [166,167].

Lynch syndrome (LS) or Hereditary Non-Polyposis Colorectal Cancer (HNPCC) is brought on by a germline heterozygous mutation in one of the four MMR genes [168]. LS is the most common inherited cancer predisposition syndrome. Germline mutations in MLH1 (42%) or MSH2 (33%), followed by MSH6 (18%) and PMS2 (7%), generally occur in a significant number of these individuals [168]. In most cases, tumors manifest themselves in the uterus and colon, but they may also manifest in other organs [168]. Patients with this condition often have a mean age lower than 45 years [169]. It is estimated that LS, an inherited genetic condition, is responsible for around two to three percent of all incidences of CRC [170].

The cumulative risk of acquiring CRC is 60–70% for men and 30–40% for women if germline mutations in MMR genes are present; the cumulative risk of developing endometrial cancer is 40–80% [170,171,172].

Compared to stage III (~12%) and stage IV (~4%) CRC, MSI is more common in stage II (~20%) than in stage III (~12%) [173,174,175,176,177]. Furthermore, it was found in two small retrospective investigations that the Egyptian population (37%) and African American population (20–45%) had higher rates of MSI malignancies [173,174,175,176,177]. It is evident that the MSI phenotype constitutes a clinically significant fraction of CRC, even if the frequency may differ throughout populations [173,174,175,176,177]. It is evident that the MSI phenotype constitutes a clinically significant fraction of CRC, even if the frequency may differ throughout populations [173,174,175,176,177].

There are some biochemical changes that are seen in families who have HNPCC, with the epigenetic suppression of MSH2 being a particularly significant example [178]. The deletion of exon 3 of the TACSTD1 gene in the germline of a heterozygous individual is the root cause of the heritable epigenetic silencing of gene MSH2 [178]. The protein known as epithelial cell adhesion molecule (EpCAM) is encoded by this gene, which is responsible for its production [178].

APC, MLH1, or PMS2 germline mutations are the source of Turcot’s syndrome, which is clinically defined by the early emergence of primary brain and colorectal tumors [179].

Roughly 15% of sporadic CRCs have MSI, most of which are associated with hypermethylation of the MLH1 promoter, which results in the loss of protein production and silencing of gene transcription [180,181].

According to the Association for Molecular Pathology, all newly diagnosed colorectal tumors should undergo MSI analysis to be divided into three subgroups: Lynch dMMR15, sporadic dMMR, and sporadic MMR-proficient [181]. The recognized clinical features and predominately right-sided localization of MSI-H CRCs predominantly affect those with female gender, advanced age, high grade, mucinous differentiation, medullary histology or signet ring, peritumoral lymphocytic infiltration, Crohn’s disease-like inflammation, diploid status, lower stage, and improved prognosis [182,183,184,185,186,187].

### 4.2. TMB

Another type of biomarker approved by the FDA for ICI therapy in cancers is the TMB, also referred to as tumor mutational load (TML) [188]. The TMB represents the total amount of modifications, or mutations, discovered in cancer cell DNA [188]. Having an understanding of the TMB could aid in therapy planning [188]. For instance, it appears that certain forms of IT may be more effective against cancers with a high number of mutations [189].

There is a variation in the link between the mutational load of tumors and the expression of PD-1/PD-L1 depending on the kind of tumor [190].

Pembrolizumab (anti-PD-1) was approved by the FDA for use in the treatment of all types of cancer due to its high TMB [191].

There have been some meta-analyses that have supported this conclusion by suggesting that the TMB is an effective predictor of response to ICI treatment [192,193]. These meta-analyses have been conducted throughout the whole cancer spectrum.

Concerns have been raised about the possibility that the FDA may have allowed Pembrolizumab for an inappropriately broad indication based on TMB [194]. Additionally, investigations that contradict each other have shown that TMB may not serve as an accurate indication of the success rate of IT [195].

## 5. Microbiota as an Immune Adjuvant for Immune Checkpoint Blockade

The microbiota, alongside PD-L1 expression, TMB, dMMR, MSI, and TME, has emerged as a crucial biomarker under continuous investigation for its role in the therapeutic response of solid cancers. It significantly contributes to the development of the intestinal immune system [2,196].

**Figure 3:**
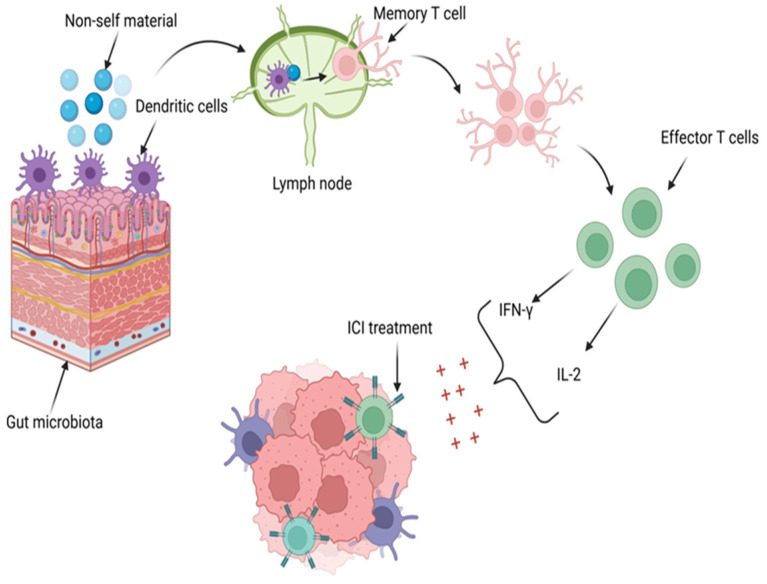
The influence of intestinal microbiota in ICI treatment. DCs in the gut microbiome identify non-self-material and present it to T cells in the lymph nodes, activating memory T cells (TCMs). These TCMs differentiate into effector T cells and proliferate, increasing levels of human IL-2 and IFN-γ. Through their primary mechanisms, IL-2 and IFN-γ enhance the effectiveness of the therapeutic response to ICIs. Created with BioRender.com (accessed on 10 August 2024).

Recent studies have demonstrated a strong association between the microbiota and the efficacy of therapeutic responses in solid tumors, including ICIs [197]. The microbiota induces an immune response against malignant cells by activating T cells and increasing the levels of human IL-2 and IFN-γ [198]. Figure 3 depicts the enhancement of treatment with ICIs through the influence of the intestinal microbiota.

The role of IFN-γ in producing a favorable response to ICI therapy is achieved through its regulation of 30 genes, stimulation of NK cell activity, enhancement of antigen presentation, and ability to increase macrophage lysosomal activity, thereby exerting antitumor and immunoregulatory effects [196,198]. Similarly, IL-2 contributes to a favorable response to ICI therapy by regulating leukocyte activity, interacting with antibodies, promoting hematopoiesis and tumor surveillance, and inducing NK cells to produce cytokines such as IFN-γ and tumor necrosis factor-α (TNF-α) [196,198].

Dysbiosis, an altered state of the gut microbiota, has been linked to chronic health conditions and cancer, but also a poor response to ICIs [16,198,199]. Dysbiosis caused by antibiotics and various diets can influence antitumor immune responses by reducing the production of deoxycholate and short-chain fatty acids [199]. Bacterial species most associated with effective ICI responses include *Bifidobacterium, Faecalibacterium, Lactobacillus,* and *Akkermansia* (HR = 2.92, 95% CI = 1.08–7.89), particularly in cases of melanoma or HCC, modulating tumor responses to checkpoint blockade IT [16,199].

A study by Gopalakrishnan et al. found significant differences in the gut microbiota of patients with melanoma. Responders to ICI treatment (CTLA-4 inhibitors and PD-1 antibodies) exhibited higher alpha diversity and an abundance of *Ruminococcaceae* bacteria (*p* < 0.01), along with enrichment of anabolic pathways and enhanced systemic and antitumor immunity [16]. The gut microbiota of responder patients was enriched with *Clostridiales*, *Faecalibacterium*, and *Ruminococcaceae* (*p* < 0.01), whereas non-responders had increased amounts of *Bacteroidales*, *Escherichia coli*, and *Anaerotruncus colihominis* (*p* < 0.01) [16].

The study also observed that responder patients had elevated levels of CD4+ and CD8+ T cells, with preserved cytokine response and a higher density of immune cells and markers of antigen processing and presentation, which can modulate antitumor immune responses [16]. In contrast, non-responders exhibited higher levels of regulatory T cells and MDSCs, along with a reduced cytokine response [16].

Unfortunately, some studies have highlighted the risk of immune-related adverse events in patients treated with ICIs. Despite *Faecalibacterium* being associated with a positive effect on ICI efficacy, an increased risk of immune-related adverse events has been observed [199]. The gut microbiome can influence the local immune system and the trafficking of bacterial peptide-primed T cells distally, potentially leading to increased toxicity from ICI treatment [199].

In a study by Xu X et al., the efficacy of PD-1 antibody IT was evaluated in CT26 tumor-bearing mice with CRC, treated with different types of antibiotics (vancomycin and colistin) and different gut microbiota compositions, compared to a control group given non-antibiotic sterile drinking water [198]. Initially, the antibiotic-treated mice showed reduced tumor growth, but this trend reversed significantly after introducing PD-1 antibody treatment [198]. Tumor growth progressed more in the vancomycin group (moderately) and the colistin group (significantly), compared to the control group, which responded favorably to the PD-1 antibody [198]. The bacterial species observed were *Bacteroides* spp. (18.76%) and *Bacteroidales* (4.6%) in the control group, *Bacteroides* spp. (20.83%), *Prevotella* spp. (7.75%), and *Bacteroidales* in the colistin-treated group, and *Prevotella* spp. and *Akkermansia muciniphila* (3.85%) in the vancomycin-treated group [198]. *Prevotella* and *Akkermansia muciniphila* were associated with a favorable response to IT due to their roles in the biosynthesis of steroid hormones, mannose, phenylpropane, beet red, and carotene [198]. In contrast, *Bacteroides* and *Bacteroidales* were linked to a poor therapeutic response through tumor signaling pathways, antigen recognition and presentation, Th17 cell differentiation, IL-17 secretion, estrogen signaling pathways, pyrimidine metabolism, and nucleic acid shearing and repair [198].

Routy et al. [14] also highlighted the positive effects of *Akkermansia muciniphila* in epithelial tumors (*p* = 0.004). Studies have shown that *Akkermansia muciniphila* enhances gut barrier function, normalizes metabolic endotoxemia and adipose tissue metabolism, improves immune responses, and decreases serum ALT, AST, pro-inflammatory cytokines, and chemokines [200,201,202].

Significant differences in metabolic and immune processes were observed when comparing the two antibiotic-treated groups. In the colistin-treated mice, abnormal T-cell glucose metabolism and chronic inflammation induced by type I diabetes mellitus, antigen processing and presentation, cancer pathways, Th 17 cell differentiation, and IL-17 signaling pathways were noted [198,202,203]. In contrast, vancomycin-treated mice showed increased glycerolipid metabolism and glycosphingolipid biosynthesis, which were associated with a more favorable response to PD-1 antibody treatment [198].

An important aspect analyzed by Xu et al. was the similarity of bacterial species before and after PD-1 antibody treatment in the three groups, indicating that the microbiota did not change post-IT [198]. The authors also noted changes in IFN-γ and IL-2 cytokines in the TME of antibiotic-treated mice, secondary to changes in gut microbiota, leading to different efficacies of PD-1 antibody treatment [198].

Recent studies have demonstrated that anti-PD-1 antibodies exhibit some efficacy in treating HCC [204,205]. Zheng et al. conducted a study analyzing eight patients with stage C HCC, according to the Barcelona Clinic Liver Cancer classification, who were treated with anti-PD-1 antibodies without any concurrent antibiotic treatment [206]. At the beginning of ICI treatment, the patients exhibited no dysbiosis, with an enrichment of Gram-positive *Firmicutes*, Gram-negative *Bacteroidetes*, and Gram-negative *Proteobacteria* in their fecal samples [206]. Throughout the treatment, patients who responded did not show significant changes in gut microbiota composition [206]. After 12 weeks, *Proteobacteria*, particularly *Escherichia coli*, became dominant in non-responding patients, while *Klebsiella pneumoniae* increased in responding patients [206]. Additionally, responding patients exhibited elevated levels of *Lactobacillus* species, *Bifidobacterium dentium*, and *Streptococcus thermophilus*, which inhibit pathogenic microorganisms [206]. They also had higher levels of *Lachnospiraceae, Ruminococcaceae* species, and *Akkermansia muciniphila*, which help maintain normal intestinal permeability and systemic immunosuppression [206]. In conclusion, Zheng et al. highlighted that the gut microbiome could serve as a biomarker for early detection of the 6-month prognosis of patients treated with PD-1 antibodies, as early as 3-6 weeks into the treatment [206].

Some studies emphasize the importance of probiotics in the intestinal flora, noting that they can increase the concentration of bacterial metabolites such as propionate and butyrate, which in turn enhance the activity of immune cells [198,207,208]. Other studies have demonstrated that the oral administration of *Bifidobacterium* can enhance the efficacy of PD-L1 antibody therapy and that oral administration of *Akkermansia muciniphila* can increase the effectiveness of PD-1 antibodies [14,209]. Table 3 summarizes the types of bacteria and their effects on the efficacy of ICI treatment responses.

Many recent studies highlight the critical importance of controlling the gut microbiota before and during treatment with PD-1 antibodies, emphasizing that maintaining a normal gut microbiota is crucial for a favorable therapeutic response [198]. Therefore, all factors that can alter the taxonomic composition, functional metagenomics, and metabolic profiles of the microbiota must be considered before starting treatment with ICIs, as these factors can significantly influence the therapeutic response to ICIs [2].

## 6. Conclusions and Future Directions

ICIs have significantly transformed cancer IT, particularly in metastatic melanoma, by improving overall survival rates [210]. These therapies target immune checkpoints like CTLA-4 and PD-1, which play essential roles in T-cell activation and maintaining immunological tolerance [211]. Despite their success, ICIs present challenges, including treatment resistance, immune-related adverse events (irAEs), and limited safety data in pediatric patients [212]. Adverse events are notably more severe with CTLA-4 inhibitors, especially at higher doses, while PD-1/PD-L1 inhibitors generally have milder toxicities, with fatigue being the most common side effect [213]. The potential for life-threatening off-target effects in children is particularly concerning [214,215]. Ongoing research is exploring novel combinations of ICIs with other therapies—such as chemotherapy, targeted agents, RT, and T-cell-based treatments—to enhance outcomes, especially in patients who do not respond favorably to ICI monotherapy [51]. Additionally, the TME plays a crucial role in treatment response, posing further complexities that require continued investigation to optimize therapeutic strategies across various cancer types [20]. Combining RT and IT may enhance their effectiveness through synergistic mechanisms, offering hope against cancers resistant to conventional treatments [216]. Additionally, photodynamic therapy (PDT), which disrupts tumor vasculature and activates the immune system, holds promise but requires carefully designed photosensitizers to work effectively in the challenging tumor microenvironment, particularly under hypoxic conditions [217].

In conclusion, the gut microbiota has emerged as a significant biomarker influencing the therapeutic response to ICIs in solid cancers. Research shows that the microbiota can enhance immune responses against tumors by activating T cells and increasing cytokine levels, such as IL-2 and IFN-γ, thereby improving ICI efficacy. However, dysbiosis—an imbalance in the gut microbiota—can lead to poor therapeutic outcomes [218]. Specific bacterial species, like *Akkermansia muciniphila* and *Bifidobacterium*, are associated with favorable responses to ICIs, while others are linked to negative effects. The gut microbiome’s composition can also predict patient prognosis early in treatment [218]. Probiotics have shown the potential to enhance the efficacy of ICIs by modulating the gut microbiota, highlighting the importance of microbiota in cancer IT.

One limitation of our study is the heterogeneity of the included trials, particularly in the definition of microbiome diversity, which may limit the generalizability of these findings. Additionally, most studies did not control for antibiotic use, which is a significant confounder in microbiome research.

Therefore, many promising future studies should consider these variables regarding anti-PD-1 and anti-CTLA-4 treatments and the improvement of their efficacy.

## Figures and Tables

**Table 1:** Classification of checkpoint inhibitors by target molecule, their brand names, their first approval dates in the USA and EU, and the first tumor type and location in which they were determined as efficient and approved for use.

Target Molecule	Checkpoint Inhibitor Name	Brand Name	First Approval FDA (USA)	First Approval EMA * (EU)	First Cancer Type Approved
PD-1	Pembrolizumab [21]	Keytruda	September 2014	July 2015	Melanoma
	Nivolumab [22] **	Opdivo	December 2014	June 2015	Melanoma
	Cemiplimab [23]	Libtayo	September 2018	June 2019	Cutaneous squamous cell carcinoma (CSCC)
	Dostalimab [24]	Jemperli	April 2021	April 2021	Endometrial cancer with dMMR/MSI-H status
	Toripalimab [25]	Loqtorzi	October 2023	ongoing	Nasopharyngeal carcinoma
PD-L1	Atezolizumab [26]	Tecentriq	May 2016	September 2017	UC
	Durvalumab [27]	Imfinzi	May 2017	September 2018	NSCLC
	Avelumab [28]	Bavencio	March 2017	September 2017	Merkel cell carcinoma
CTLA-4	Ipilimumab [29] **	Yervoy	March 2011	July 2011	Melanoma
	Tremelimumab [30]	Imjudo	October 2022	December 2022	HCC (in combination)

* European Medicines Agency (EMA). ** Nivolumab + Ipilimumab in combination approved by the FDA in April 2018 and EMA in October 2018 for RCC [31].

**Table 2:** Predictive biomarkers approved or being studied for specific treatment of different types of cancer with KRAS mutations.

Predictive Biomarker	Cancer Type	Drugs
KRAS G12C [117]	NSCLC, pancreatic ductal adenocarcinoma and CRC	Sotorasib, Adagrasib (approved)
	Advanced or metastatic solid tumors	GDC-6036—monotherapy or in combination with Cetuximab, Atezolizumab, Bevacizumab
	Advanced solid tumors	JDQ443—monotherapy or in combination with TNO155 or Tislelizumab
	Still in phase I/II studies	D-1553, LY3537982, JAB-21822
KRAS G12D [117]	Still in preclinical studies	MRTX1133—long half-life, potency, and antitumor activity
KRAS G12D, G12V, G13D, and G12C [117]	Still in phase I study in patients with metastatic NSCLC, CRC, or pancreatic adenocarcinoma	V941—lipid nanoparticle-formulated mRNA-based cancer vaccine, potential immunostimulatory and antineoplastic activities
NRF2-KEAP1 axis [116]	Estrogen receptors (ERs) + metastatic breast cancer	SFX-01—safe and efficacious when used in combination with aromatase inhibitors, Tamoxifen and Fulvestrant
NRF2 inhibitors [118]	Still in studies—lymphocytic leukemia tumor cells and many types of solid cancer cells	Brusatol
	Still in preclinical models—NSCLC cells with KEAP1 mutations	ML385—combined with Doxorubicin, Taxol, or Carboplatin
	Phase 2 clinical trials for cancer	Halofuginone—inhibitor of collagen type I synthesis and of prolyl-tRNA synthetase
	Phase 2 clinical trial for bladder cancer treatment	HT-100—oral analog of halofuginone

**Table 3:** The effect of different types of bacteria on ICI treatment response.

Favorable Response	Poor Response
*Clostridiales* [16]	*Bacteroidales* [16,198]
*Faecalibacterium* [16]	*Escherichia coli* [16,206]
*Ruminococcaceae* [16]	*Anaerotruncus colihominis* [16]
*Prevotella* [198]	*Bacteroides* [198]
*Akkermansia muciniphila* [198]	
*Bifidobacterium* [199]	
*Lactobacillus* [199]	
*Klebsiella pneumoniae* [206]	
*Streptococcus thermophiles* [206]	
*Lachnospiraceae* [206]	

## Abbreviations

APCs	antigen-presenting cells
B2M	beta-2-microglobulin
CRC	colorectal cancer
CTLA-4	cytotoxic T-lymphocyte-associated protein 4
DDR	DNA damage response
DNA	deoxyribonucleic acid
dMMR	defective DNA mismatch repair
EMA	European Medicines Agency
ER	estrogen receptor
EU	European Union
EpCAM	epithelial cell adhesion molecule
FDA	Food and Drug Administration
GITR	glucocorticoid-induced TNFR-related protein
HCC	hepatocellular carcinoma
HER2	human epidermal growth factor receptor 2
HLA-DR	human leukocyte antigen-DR isotype
HNPCC	Hereditary Non-Polyposis Colorectal Cancer
ICI	immune checkpoint inhibitor
IFN-γ	interferon-gamma
Ig	immunoglobulin
IL-10	interleukin 10
irAEs	immune-related adverse events
IT	immunotherapy
ITIM	immunoreceptor tyrosine-based inhibitory motif
JAK1/2	Janus kinases
KEAP 1	Kelch-like ECH-associated protein 1
KRAS	Kirsten rat sarcoma viral oncogene homolog
LAG-3	lymphocyte activation gene-3
LS	Lynch syndrome
MDSCs	myeloid-derived suppressor cells
MET	mesenchymal–epithelial transition
MHC	major histocompatibility complex
MSI	microsatellite instability
MSI-H	microsatellite instability-high
NK	natural killer
NRF2	nuclear factor erythroid 2-related factor 2
NSCLC	non-small cell lung cancer
NTRK	neurotrophic tyrosine receptor kinase
PD-1	programmed cell death 1
PD-L1	programmed cell death ligand 1
PDT	photodynamic therapy
PMBCL	primary mediastinal large B-cell lymphoma
POLE	polymerase epsilon
RCC	renal cell carcinoma
RET	rearranged during transfection
RT	radiotherapy
SCLC	small cell lung cancer
SHP-1, SHP-2	protein tyrosine phosphatase 1 and 2
STK11	serine/threonine kinase 11
STK11/LKB1	STK11 gene codes for liver kinase B1
TAMs	tumor-associated macrophages
TCMs	memory T cells
TCR	T-cell receptor
TGF-β	transforming growth factor beta
Th	helper T cell
TIGIT	T-cell immunoglobulin and ITIM domain
TILs	tumor-infiltrating T cells
TIM-3	T-cell immunoglobulin and mucin domain-containing protein 3
TMB	tumor mutational burden
TML	tumor mutational load
TME	tumor microenvironment
TNBC	triple-negative breast cancer
TNF-α	tumor necrosis factor-α
TPEX	exhausted T cells and their precursors
TRM	tissue-resident memory T cells
Tregs	regulatory T cells
UC	urothelial carcinoma
USA	United States of America
VEGF	vascular endothelial growth factor
VISTA	V-domain Ig suppressor of T-cell activation
